# Rapid Microfluidic Assay for the Detection of Botulinum Neurotoxin in Animal Sera

**DOI:** 10.3390/toxins8010013

**Published:** 2016-01-04

**Authors:** Lmar Babrak, Alice Lin, Larry H. Stanker, Jeffery McGarvey, Robert Hnasko

**Affiliations:** 1 Produce Safety & Microbiology Research Unit, United States Department of Agriculture, Agricultural Research Service, 800 Buchanan St, Albany, CA 94710, USA; lmar.babrak@ars.usda.gov (L.B.); alice.lin@ars.usda.gov (A.L.); 2 Foodborne Toxin Detection and Prevention Unit, United States Department of Agriculture, Agricultural Research Service, 800 Buchanan St, Albany, CA 94710, USA; larry.stanker@ars.usda.gov (L.H.S.); jeffery.mcgarvey@ars.usda.gov (J.M.)

**Keywords:** toxin, botulinum neurotoxin (BoNT), botulism, microfluidic, immunoassay, rapid detection, animal serum, diagnostic

## Abstract

Potent Botulinum neurotoxins (BoNTs) represent a threat to public health and safety. Botulism is a disease caused by BoNT intoxication that results in muscle paralysis that can be fatal. Sensitive assays capable of detecting BoNTs from different substrates and settings are essential to limit foodborne contamination and morbidity. In this report, we describe a rapid 96-well microfluidic double sandwich immunoassay for the sensitive detection of BoNT-A from animal sera. This BoNT microfluidic assay requires only 5 μL of serum, provides results in 75 min using a standard fluorescence microplate reader and generates minimal hazardous waste. The assay has a <30 pg·mL^−1^ limit of detection (LOD) of BoNT-A from spiked human serum. This sensitive microfluidic BoNT-A assay offers a fast and simplified workflow suitable for the detection of BoNT-A from serum samples of limited volume in most laboratory settings.

## 1. Introduction

The Botulinum neurotoxins (BoNTs) are produced by the ubiquitous, spore forming, anaerobic bacterium, *Clostridium botulinum*. These potent neurotoxins are biologically active at very low doses resulting in rapid flaccid muscle paralysis that can be fatal [1,2]. There are seven distinct BoNT serotypes (A–H), with BoNT-A and -B the most commonly associated with disease [3,4]. The susceptibility of animals to botulism intoxication by ingestion of contaminated foods and its potential use as a biothreat agent necessitates rapid detection strategies to limit foodborne contamination and morbidity [3,5,6].

Currently, a mouse bioassay is the only FDA-approved method for the detection of BoNT contamination. Although this bioassay is sensitive, it is performed by only a few specialized laboratories and requires ~4 days for the confirmation of results [7,8]. Alternate in vitro assays have been developed to address the need for more robust testing capabilities from a variety of substrates and settings [9,10,11,12]. Given the potency of the BoNTs, these assays must achieve a high degree of sensitivity and yield rapid results to prevent contamination from entering the food supply and provide actionable options in clinical settings.

No single assay will likely meet all the challenges of BoNT detection as different substrates and environments will require distinct strategies to best meet end-user requirements. In this manuscript, we describe the development of a rapid, microfluidic, double sandwich immunoassay suitable for the sensitive detection of BoNT-A from low volume animal sera. We take advantage of microfluidic technology that enables faster reaction kinetics, requires minimal sample and reagent volumes, generates reduced hazardous waste, and uses a standard 96-well microplate format that is compatible with ELISA plate readers.

## 2. Results

### 2.1. Microfluidic Assay Design

**Figure 1:**
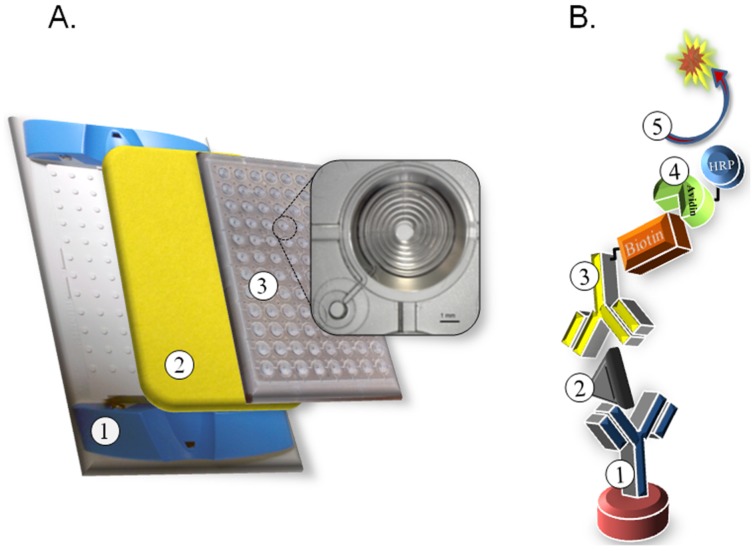
Schematic of microfluidic plate assembly and BoNT-A double sandwich assay. (**A**) Plastic plate holder ①, absorbent pad ②, clear 96-well microfluidic plate ③ with inset showing a single microfluid well with channel geometry; (**B**) Illustration depicting biological components in BoNT double sandwich assay. ① immobilized capture antibody (F1-2 mAb), ② antigen (BoNT-A), ③ biotinylated detection antibody (F1-51 mAb), ④ avidin conjugated to horseradish peroxidase (HRP), ⑤ QuantaRed substrate with fluorescent signal generation (570–585 nm ex/em).

To develop our BoNT-A microfluidic assay, we utilized clear 96-well microfluidic plates where each well consists of a tapered 200 × 200 μm spiral channel with a standard microplate footprint, height, and well spacing (Siloam Biosciences, Cincinnati, OH, USA). When compared to a conventional flat-bottom microplate, the microfluidic well provides an increased surface area, faster reaction kinetics, and only 5 μL of sample volume. Liquid added to the tapered top of each well flows passively through each microfluid channel mediated by capillary action toward a plastic backed absorbent pad held in place on the back of the plate using a plate holder (Figure 1A). The immunoassay design is a double sandwich format where a captured anti-BoNT-A monoclonal antibody (F1-2) is immobilized in the microfluid channel, sample is applied, and detection is mediated by addition of a biotinylated anti-BoNT-A monoclonal antibody (Btn-F1-51) followed by avidin conjugated to horseradish peroxidase (avidin-HRP) and the signal is resolved using chemifluorescent QuantaRed substrate (ThermoFisher Scientific, Waltham, MA, USA) detected using ~570/585 nm excitation/emission filters (Figure 1B).

The familiar 96-well format allows for the use of conventional multi-channel pipettes for liquid handling, easy sample replication, minimal reagent consumption, and reduced biohazardous waste. The assay requires only 5 μL per well of sample and reagent buffers which is 20-fold less volume than used in a conventional microplate ELISA. The geometry of each well provides about 50× more surface area-to-volume for binding of the capture antibody and increases the reaction kinetics to significantly reduce incubation times as compared to a conventional ELISA performed in flat bottomed microplates. Table 1 lists the steps to perform the microfluidic BoNT-A assay, the associated reagents, volumes, and incubation times.

**Table 1:** BoNT-A microfluidic assay steps. Sequential steps with associated reagent, volume and incubation times. mAb = monoclonal antibody, Btn = biotin, sAv-HRP = streptavidin − horseradish peroxidase.

Assay Steps	Time (min)	Reagent	Volume (µL)
Immobilized Capture mAb	5	BoNT mAb F1-2	5
Wash	5	OptiWash	5
Block	5	OptiBlock	5
Toxin	5	BoNT-A	5
Wash	5	OptiWash	5
Detector mAb	5	Btn BoNT mAb F1-51	5
Wash	5	OptiWash	5
Reporter	5	sAv-HRP	5
Wash	20	OptiWash	60
Substrate	15	QuantaRed	10
	75 min		110 µL

### 2.2. BoNT Microfluidic Immunoassay Parameters

**Figure 2:**
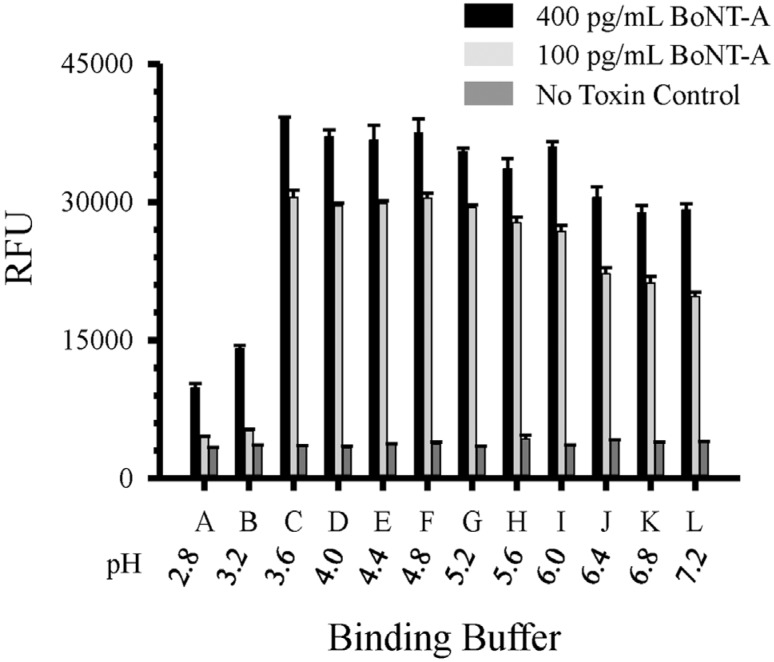
Comparison of buffers (A–L) for antibody immobilization on microfluidic plate. Capture BoNT-A F1-2 mAb (20 μg·mL^−1^) diluted in binding buffers A–L (pH 2.8–7.2) and double sandwich assay performed with biotinylated F1-51 (1 μg·mL^−1^) for detection of BoNT-A toxin. Data represents a mean of three replicates with standard deviations. RFU = relative fluorescent units.

The BoNT-A monoclonal antibody pair (F1-2 and F1-51) have been previously characterized and shown to be effective in a double sandwich ELISA and lateral flow assay [9,10,13]. The first step in building the double sandwich immunoassay is the immobilization of the capture antibody (F1-2) to a plastic surface. Standard microplates are produced from hydrophobic polystyrene (PS) which are easily modified after manufacture to alter the surface chemistry to promote biomolecule interaction by passive adsorption through hydrophobic and ionic interactions [14]. The stable immobilization of purified antibody to high-binding PS can usually be accomplished in a simple carbonate/bicarbonate buffer (pH 9.5). However, unlike a conventional microplate well, the reaction rate of protein adsorption in the microfluid channel is strongly affected by the pH of the binding buffer. Toward that aim we evaluated 12 different antibody binding buffers (citric acid-sodium phosphate buffer A–L pH 2.8–7.2; +0.4 pH units/letter) to determine the optimal buffer for antibody immobilization and downstream assay performance (Figure 2). These data demonstrate optimal buffers for use with our anti-BoNT mAb pair and buffer C (pH 3.6) was chosen for all subsequent experiments.

A typical microplate sandwich ELISA is well adapted to automated liquid handling systems [15]. These assays often use 100 μL well volumes with extensive washing between assay steps and require incubation times that result in assay times >5 h to complete. In contrast, the small volume (5 μL) and precise well loading required on the microfluidic plate is better suited for manual liquid dispensing as care must be taken to ensure no bubbles enter the microfluid channel. Although less automated, the rapid 5 min incubation times at each step reduces total assay time to ~75 min. Moreover, the small volumes used in the microfluidic assay reduce total reagent costs and the liquid biohazardous waste generated is completely contained on a single disposable pad. Only a 0.11 mL of waste is generated by a microfluidic well as compared to >3.1 mL generated by a microplate well. This difference is significant, with >300 mL of biohazardous waste generated per microplate assay that may require expensive disposal options.

The standard dimension of the microfluidic plate allows the use of existing laboratory plate readers for detection. The chemifluorescent QuantaRed substrate (~570/585 nm ex/em maxima) allows for both colorimetric and fluorescent measurements. The reaction product can be measured at 576 nm on a colorimetric plate reader or using non-overlapping filter sets at 530–575 nm for excitation and 585–630 nm for emission for fluorescent detection. The instrument settings will affect the performance of the QuantaRed substrate with adjustments in filter bandpass range, instrument gain settings, and photomultiplier tube voltage influencing the signal intensity. The instrument settings will define the integrated signal generated and consequently fluorometric units are defined as relative fluorescent units (RFU).

### 2.3. BoNT-A Microfluidic Immunoassay Performance

**Figure 3:**
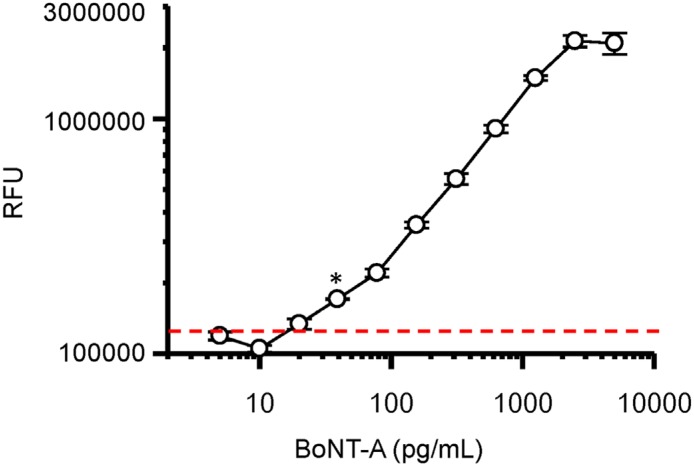
Dose-dependent detection of BoNT-A holotoxin by double sandwich microfluidic assay. Dashed red line indicates three standard deviations above no toxin background. * Indicates limit of detection (LOD) of BoNT-A at ~32 pg·mL^−1^. Circles represent a mean of three replicates with bars indicating standard deviation. RFU = relative fluorescent units.

Optimal concentration of BoNT-A capture mAb F1-2 (20 μg·mL^−1^) was diluted in binding buffer C (pH 3.6) and immobilized in the microfluid channel. After well blocking, decreasing concentrations of BoNT-A holotoxin (5000–5 pg·mL^−1^) diluted in blocking buffer were added and fluorescent detection determined following sequential addition of biotinlyated anti-BoNT mAb F1-51 (1 μg·mL^−1^), avidin-HRP, and the QuantaRed substrate (Figure 3).

These data establish a 2-log dynamic range of the BoNT-A microfluidic assay from 2000 to 20 pg·mL^−1^ with a limit of detection (LOD) between 20 and 40 pg·mL^−1^. This microfluidic assay was compared to our conventional chemiluminescent microplate double sandwich ELISA [10,13], using the same antibodies and BoNT-A samples, with both assays resulting in similar detection sensitivities and dynamic range (data not shown). The microfluidic assay used 20-fold less sample volume, but required more concentrated antibody samples.

**Figure 4:**
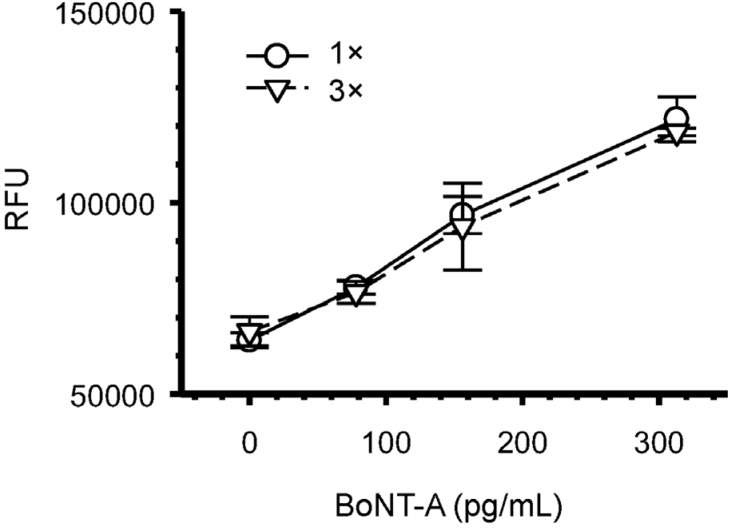
Repeated applications of BoNT-A sample to microfluidic well does not increase the assay sensitivity. The addition of a single 5 μL BoNT-A (1×) was compared three sequential 5 μL BoNT-A (3×) samples added to each microfluidic well. Symbols represent the mean for each BoNT-A dilution from three replicates with bars indicating standard deviations. RFU = relative fluorescent units.

In an attempt to improve the sensitivity of the microfluidic assay, we evaluated the use of a poly-avidin-HRP for detection. This resulted in increased background fluorescence that decreased our signal-to-noise, reducing both the dynamic range of the assay and the LOD (data not shown). Similar results were obtained with the use of a chemiluminescent substrate for signal generation (data not shown). Figure 4 compares the results obtained after repeated addition of BoNT-A sample (3×) to that of a single pass (1×) to each microfluidic well. These experiments show no difference in the detection sensitivity of the assay after the cumulative addition of the BoNT-A toxin.

### 2.4. Detection of BoNT-A in Animal Sera by Microfluidic Immunoassay

**Figure 5:**
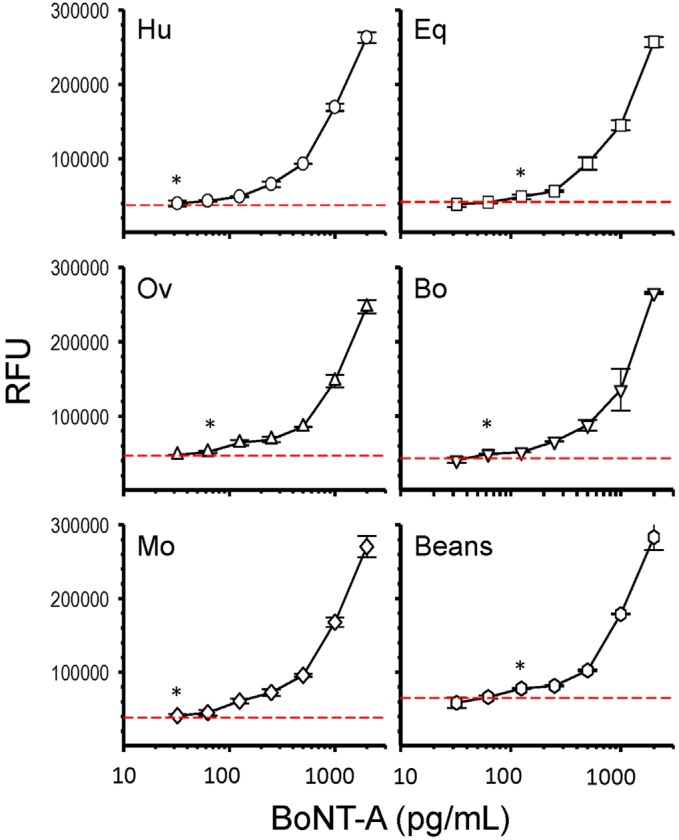
Dose-dependent detection of BoNT-A in animal sera by microfluidic immunoassay. BoNT-A holotoxin was spiked into animal sera: Human (Hu), equine (Eq), ovine (Ov), bovine (Bo), and mouse (Mo), and the juice from canned green beans, at seven concentrations between 2000 and 32 pg/mL. BoNT-A was detected in the serum sample by a double sandwich microfluidic immunoassay and the limit of detection (LOD) defined (*). The dotted red line indicates the mean assay background (serum without toxin) plus three times the standard deviation. Each data point (symbol) represents a mean of three independent sample replicates with bars indicating the standard deviation. RFU = relative fluorescent units.

Animal serum represents a useful sample for confirmation of BoNT intoxication [8]. Serum is a complex matrix and different components can interfere with an assay to limit detection sensitivity. We spiked decreasing concentrations of the BoNT-A holotoxin into sera to determine the sensitivity of our microfluidic assay from different animal species in three independent experiments (Figure 5). First the flow of undiluted serum through the microfluidic channel was confirmed. Serum from each species were initially spiked with BoNT-A at 100 ng·mL^−1^ which was then used to serially dilute serum samples used in the assay titration. The microfluidic assay was useful for the detection of BoNT-A from all five of the animal sera tested and LODs were defined by the lowest detectable BoNT-A dilution that was above assay background established as the mean of serum without toxin plus three times the standard deviation (Table 2). Human and mouse sera had the least assay interference with LODs of ≤32 pg·mL^−1^. The LOD achieved with bovine and ovine sera were ≤63 pg·mL^−1^ whereas equine sera had the most assay interference and a ≤125 pg·mL^−1^ LOD. The juice from canned beans was also evaluated and despite a higher assay background an LOD of ≤125 pg·mL^−1^ was defined.

**Table 2:** Limit of detection (LOD) for BoNT-A in serum samples by microfluidic immunoassay. BoNT-A spiked serum samples (triplicate) were titrated and LOD concentration (pg·mL^−1^) determined by selecting the first dilution that was greater than the mean of assay background plus three standard deviations (serum without toxin). Canned beans represent data obtained from spiked juice after centrifugation.

Serum	Limit of Detection (≤pg/mL)
Human	32
Mouse	32
Bovine	63
Ovine	63
Horse	125
Canned Beans	125

## 3. Discussion

Botulism is most commonly acquired by ingestion of contaminated food or in unmanaged wounds [1,3]. In suspect cases of BoNT intoxication a serum diagnostic test can facilitate treatment to reduce morbidity and mortality. Infants represent a high risk group accounting for the majority of clinical incidents [16]. When initiating diagnostic test panels the patient’s serum sample is often partitioned to accommodate the need for different target tests. This can limit the amount of available sample for any one test. The 5 μL sample requirement for use in our microfluidic BoNT-A assay provides a reasonable option. This low volume capability affords the evaluation of sample replicates to improve diagnostic confidence. The sensitivity of this assay is within a clinically relevant range [17] and is comparable to those achieved with a conventional microplate ELISA [10].

Although the mouse BoNT bioassay is extremely sensitive, it is often impractical and requires 2–4 days to obtain results [8]. BoNT ELISA assays provide a good alternative but require sample dilution and most will take >5 h to complete. Our microfluidic BoNT-A assay uses the same laboratory instrumentation and workflow to perform a standard ELISA to achieve equivalent results in 75 min with less sample and biohazardous waste. Indeed, the microfluidic assay consumes significantly less reagents and the liquid waste, reduced to a single absorbent pad, which will reduce assay cost and simplify disposal.

The 96-well format of the microfluidic plate offers a familiar laboratory assay platform that is amenable to automation, increased throughput, and sample replicates. Microfluidics is an emerging technology designed for the controlled movement of small fluid volumes through a microchannel network [18,19]. In principle, microfluidic platforms offer the potential for a portable, simple-to-use, and inexpensive lab-on-chip device for rapid multi-analyte detection of small volume samples [20,21,22]. Indeed, a multitude of customized microfluidic solutions have been shown effective for analyte detection as R&D tools, but few have translated into successful commercial products adopted by end-users [18]. This in part reflects the high cost of R&D, challenges associated with fabrication, material composition, and sample application. The Optimizer microfluidic plate allows assay developers a flexible platform to identify suitable reagents and evaluate their performance in microfluid channels across parameters of speed, sensitivity, and sample volume.

The BoNT microfluidic assay demonstrated good performance characteristic with a high degree of reproducibility and dynamic range with no observable hook effect. There remains a need for the evaluation of clinically relevant samples to determine the usefulness of this assay in clinical settings. We are currently working to transfer BoNT serotype specific immunoassays to this microfluidic platform which would allow identification and differentiation of any of the BoNTs from a given animal sera. These commercially available microfluidic plates provide a robust platform to establish immunoassays for analyte detection where sample volumes are limited and rapid results desired.

## 4. Conclusions

We have developed a rapid and sensitive microfluidic immunoassay for the detection of BoNT-A from animal sera. This assay requires only 5 μL of serum, yields clinically relevant results in 75 min, and generates minimal biohazardous liquid waste that is contained in a single disposable absorbent pad. This economical BoNT microfluidic assay provides a 96-well plate format for use with standard laboratory equipment to evaluate animal sera and other potentially contaminated substrates.

## 5. Experimental Section

### 5.1. Reagents and Materials

All reagents purchased were of the highest grade possible. BoNT-A serotype specific monoclonal antibodies (F1-2 and F1-51) were previously generated and characterized [13,23,24]. The BoNT-A holotoxin was purchased from List Biological Laboratories Inc., Campbel, CA, USA. Human and mouse serum were purchased from Sigma-Aldrich (St. Louis, MO, USA), equine and ovine sera were purchased from Gibco (Billings, MT, USA), and bovine serum purchased from Biowhittaker (Walkersville, MD, USA). Canned beans were purchased from a local food pantry. Microfluidic plates (Optimiser) and associated buffers were purchased from Siloam Biosciences (Cincinnati, OH, USA). The microfluidic plate conforms to standard 96-well plate dimensions established by the Society for Biomolecular Screening (SBS, Danbury, CT, USA). Well diameter and plate location meet SBS format requirements with each consisting of a shallow hydrophilic tapered well leading to a hollow 200 × 200 μm coiled channel. The geometry of each well is designed to facilitate fluid flow into and through the channel by capillary action toward a plastic-backed absorbent pad, which is in contact with the exit port of each well, held in place on the underside of the plate with a plate holder (Figure 1A). QuantaRed substrate was purchased from Pierce (Madison, WI, USA).

### 5.2. Microfluidic BoNT-A Immunoassay Methodology

The assay is performed at room temperature and liquid dispensed using a P20 micropipettor in a biosafety cabinet. The absorbent pad is placed in the plate holder and the 96-well microfluidic plate snapped into place (Figure 1A). Sequentially to each microfluid well: 5 μL anti-BoNT-A F1-2 mAb diluted in buffer C (pH 3.6 at 20 μg·mL^−1^) 5 min, 5 μL wash buffer 5 min, 5 μL block 5 min, 5 μL sample 5 min, 5 μL anti-BoNT monoclonal antibody F1-51 (1 μg·mL^−1^) diluted in blocking buffer 5 min, 5 μL wash buffer 5 min, 5 μL streptavidin-HRP (0.5 mg·mL^−1^; 1:150) in block buffer 5 min, two × 30 μL wash buffer 10 min each, 10 μL QuantaRed substrate 15 min. After incubation with the substrate, the microfluidic plate is removed from the plate holder, the bottom of the plate is wiped, and fluorescence measurements are obtained for each well using a Perkin-Elmer Victor X3 plate reader with a 530/595 ± 20 nm excitation and emission filter set.

### 5.3. BoNT-A Sera Spike and Dilution Series

A BoNT-A holotoxin stock was diluted to 1 μg/mL in saline to make a working solution. A pool of sera representing each animal species was spiked initially to 100 ng·mL^−1^ from the BoNT-A working solution and serial dilutions using the corresponding animal sera were used to make the dilutions used in the assay titration. The juice from canned beans was centrifuged at 16,000× *g* for 10 min and the supernatant spiked to 100 ng·mL^−1^ and serial dilutions performed using the supernatant for use in the assay.

### 5.4. Statistics

Each data point was performed in triplicate and represents a mean ± standard deviation of relative fluorescent units (RFU). The assay background was defined in the absence of toxin for each substrate. The limit of detection (LOD) was defined for each assay as the first concentration of BoNT-A above the mean of the assay background plus three standard deviations.
